# Why Cohen’s *Kappa* should be avoided as performance measure in classification

**DOI:** 10.1371/journal.pone.0222916

**Published:** 2019-09-26

**Authors:** Rosario Delgado, Xavier-Andoni Tibau

**Affiliations:** 1 Department of Mathematics, Universitat Autònoma de Barcelona, Campus de la UAB, Cerdanyola del Vallès, Spain; 2 Advanced Stochastic Modelling research group, Universitat Autònoma de Barcelona, Campus de la UAB, Cerdanyola del Vallès, Spain; UCLA, UNITED STATES

## Abstract

We show that Cohen’s *Kappa* and Matthews Correlation Coefficient (MCC), both extended and contrasted measures of performance in multi-class classification, are correlated in most situations, albeit can differ in others. Indeed, although in the symmetric case both match, we consider different unbalanced situations in which *Kappa* exhibits an undesired behaviour, i.e. a worse classifier gets higher *Kappa* score, differing qualitatively from that of MCC. The debate about the incoherence in the behaviour of *Kappa* revolves around the convenience, or not, of using a relative metric, which makes the interpretation of its values difficult. We extend these concerns by showing that its pitfalls can go even further. Through experimentation, we present a novel approach to this topic. We carry on a comprehensive study that identifies an scenario in which the contradictory behaviour among MCC and *Kappa* emerges. Specifically, we find out that when there is a decrease to zero of the entropy of the elements out of the diagonal of the confusion matrix associated to a classifier, the discrepancy between *Kappa* and MCC rise, pointing to an anomalous performance of the former. We believe that this finding disables *Kappa* to be used in general as a performance measure to compare classifiers.

## Introduction

Classification is one of the cornerstones of Supervised Machine Learning. In parallel to the development of different methodologies that allow the construction of classifiers, the evaluation process of the classifiers to compare them, and the choice of the best among those available, has caught the attention of researchers.

Introduction of an adequate performance measure for classifiers is a subject no yet closed up to date (see [1]-[3]), and different metrics have been introduced. Some measures are naturally introduced in the binary case, such as Accuracy, Sensitivity, Specificity and Area Under the ROC Curve (AUC), among others, but not all of them can be well extended to the multi-class setting.

One of the ones that does is Accuracy (i.e. the fraction of well-predicted cases over the total), which seems the most natural measure and has been used for decades. Notwithstanding, Accuracy is not an effective measure since, among other things, it does not take into account the distribution of the misclassification among classes nor the marginal distributions. Other more subtle measures have been introduced in the multi-class setting to address this issue, improving efficiency and class discrimination power.

We will focus our attention in Matthews Correlation Coefficient (MCC) and Cohen’s *Kappa*. The former was introduced in the binary setting by Matthews ([4]), and generalized to the multi-class case in [5], being commonly used as a reference performance measure, especially for unbalanced data sets, in different fields as, for example, bioinformatics (see [5]-[7]). On the other hand, *Kappa* is a traditional measure originally designed as a measure of agreement between two judges, based on the Accuracy but corrected for chance agreement. At present, its use is not simply limited to medicine or psychology (see for instance, [8] and [9]), but is a measure widely used in other fields as ecology ([10] and [11]), neuroscience ([12]) or machine learning, where it is used to evaluate the agreement between the actual and the assigned classes by a classifier. In the classification literature, the discussion on *Kappa* is most focused on its suitability compared to other classifiers; for example, in [1] *Kappa* has been considered jointly with 17 other performance metrics in several scenarios.

It is not an overstatement to say that *Kappa* is one of the most widespread measures and of use in several fields and disciplines. Nevertheless, some authors, including the introducer of *Kappa* statistic himself, Jakob Cohen, alerted that *Kappa* could be inadequate in different circumstances, specifically when an imbalance distribution of classes is involved, i.e. the marginal probability of one class is much more (or less) greater than the others (leaving aside the literature below, on which we will deal more closely, see also [13]-[17]). According to them, some problems arise in such situations because it is not clear how the hypothetical probability of chance agreement should be defined. In [18] and [19], the so-called *Kappa paradox* is described. Roughly speaking, *Kappa paradox* arises since for a fixed agreement between judges, the *Kappa* statistic penalizes judges with similar marginals compared with judges with different ones. The authors show several examples where this happens.

This same obstacle is extensively studied in [20]-[22]. In the later, two separate causes of the *paradox* are considered; (1) the *prevalence paradox* arises from the fact that when the hypothetical probability of chance agreement among raters is high, even high values of the relative observed agreement (which is identical to Accuracy) produce low values of *Kappa*, and (2) the *bias paradox*, which is the consequence of the fact that imbalanced marginal distributions produce higher scores of *Kappa*. The authors claim that reporting a single agreement coefficient makes interpretation and comparison difficult. Hence, they suggest a corrected version of *Kappa* for *bias* and *prevalence* (PABAK), which should be used together with *Kappa*.

Similar conclusions emerge from [23], where the authors claim that *Kappa* is a relative measure of agreement, which is an inadequate characteristic for assessing in a clinical setting, specifically if a high agreement among experts leads to lower values of *Kappa*. Instead, they suggest using *the proportion of specific agreement* ([24]), which divides the agreement into a positive and a negative rate, allowing professionals to have an absolute measure and at the same time, information about the marginal distributions. Regarding the effect on estimation of the chance agreement, Albatine et al. ([25]) analysed 28 different similarity measures for clustering purposes; they suggest adding a correction for chance, in a specific family of coefficients, which makes some of them equivalent, regardless of how expectations are calculated. This work is extended by Warrens in [26], where more in-depth analysis is presented and several indices are generalized: Cohen’s kappa ([27]), Scott’s pi ([28]), Mak’s rho([29]), Goodman and Kruskal’s lambda ([30]), and Hamann’s eta ([31]).

On the other hand, there are several authors that defend that *Kappa* is a useful measure of agreement, when its limitations are taken into account. For example, in [32] the authors defend the use of *Kappa* in a previous study, and warn that it is a useful measure if marginal distributions are considered. A similar conclusion was reached in [33], where it is said that although *Kappa* is not suitable in certain circumstances, it is better than the raw proportion. In [34] the work of [22] expands and the *Kappa* pitfalls are explained for the agreement between judgments, concluding that if it is used and interpreted properly, the *Kappa* coefficient provides a valuable information. As in previous works, they propose to use corrected versions of the coefficient as well. In [16] the author argues that in the case of dichotomous variables, *Kappa* is satisfactory (although it is not for other cases); as we show in the present work, even in the binary case, *Kappa* can exhibit unexpected behaviour. Finally, there are some authors ([34]) who do not agree with the use of weighted versions of the statistics as PABAK, and suggest select the marginal distributions to be similar.

In general, the use of *Kappa* is not only extended but accepted, and its pitfalls are overcome by considering the marginal distributions and using weighted alternatives, as, for example the one suggested by Cohen ([15]), PABAK or other alternatives ([35] and [36]).

Despite the vast amount of existing literature, in the field of medicine and psychology, pointing out the threats of *Kappa*, when Classification Machine Learning methods experimented their boom Cohen’s *Kappa* was introduced as a reliable performance metric. Actually it is incorporated in the most extended software packages, such as *SciKit Learn* [37] for Python, and *Caret* [38] for R. What is more, in recent studies such as [39]-[42] and [12], *Kappa* is still used as if it were a reliable performance metric. In fact, the literature reviewed recognizes the difficulty of clinical professionals in interpreting *Kappa* because it is a relative measure, that is, *Kappa* itself is not enough to know if two professionals agree or disagree. This does not seem to be a problem in machine learning classification because the ground-truth is always compared with different methods in the same condition of marginal distributions. Therefore, it can be argued that we are not interested in the value of *Kappa* itself (as are the clinicians), but in the difference of the classifying pairs ground-truth, so *Kappa* is a reliable metric for this task. However, the reality is that this is not always the case. As we show, there are scenarios in which, given the same ground-truth, a better classifier can obtain a lower value of *Kappa*. It is important to mention that some authors also highlight the problems associated with *Kappa* when it is used as a performance metric in classification (see for instance [43]-[45]), although they do not perform an exhaustive analysis like the one presented here.

Clearly, marginal distributions seem to play a key role in the problems surrounding *Kappa*. However, there is a lack of a consistent and satisfactory description of the cases in which the unwanted behaviour of *Kappa* appears, and how this affects its use as a performance metric for classification.

In our paper, we deepen the study of the pitfalls discussed above by analysing in detail the unwanted behaviour of *Kappa* from a novel perspective. Our point of view is the identification of situations in which discrepancies in its behaviour, with respect to that of MCC, become evident, going in the opposite direction. Indeed, we study varied scenarios of misclassification in settings with different marginal probabilities of the categories, and how this scenarios affect the statistics *Kappa* and MCC, by analysing both the asymmetry and the entropy of the confusion matrix. Considering *Kappa* as a relative measure of agreement, we provide a mathematical framework to understand the associated problems with it when dealing with extreme unbalanced marginal distributions, which is frequent in machine learning problems.

Our goal is to present a systematic study, both analytical and by means of empirical experimentation, to compare the two performance measures. For that, we investigate the similarities and differences in the behaviour of MCC and *Kappa* in different scenarios. In some of them, they are strongly correlated, and we show some mathematical relations and study some limit cases. But in others, they exhibit very different behaviour, being that of *Kappa* contrary to common sense, to the point that we join the detractors of its use for the assessment of classifiers. This paper is an attempt to shed some light on the identification of the latter.

The paper is organized as follows: first, we introduce some definitions and state some notations. Next, we prove that if the confusion matrix, which allows visualization of the performance of a classifier, is symmetric, then *Kappa* and MCC coincide. Each column in the confusion matrix represents the cases in any predicted class, while each row represents the cases in any actual class. In the sequel, we study in some detail the binary case, in which classes are named “positive” and “negative” and the confusion matrix has a general form $\left(\begin{array}{cc} a & b\\ c & d \end{array}\right)$, where *a* = *true positive*, *b* = *false negative*, *c* = *false positive* and *d* = *true negative*, splitting the study according to whether *c* = 0, the scenario in which *Kappa* has a behaviour consistent with that of MCC, and *c* > 0, in which the opposite happens. For each of these cases, we consider particular sub-cases and we deepen in their study. We also consider a pathological multi-class unbalanced situation, in which one of the classes is much more common than the others, and it is mainly misclassified (family of confusion matrices *Z*_*A*_ introduced in [2]). We also perform empirical experimentation in dimension 3, considering some families of confusion matrices, and finish with a few concluding words.

## Definitions and notations

Given a generic matrix *M*, let *M*^*T*^ denote its transpose, that is, the matrix obtained from *M* by interchanging columns and rows. The same notation applies to vectors, which by default are column vectors. We say that matrix *Q* is equivalent to *M*, and denote it by *Q* ≡ *M*, if *Q* can be obtained from *M* by multiplying it by a positive constant.

### Classification

Classification consists of assigning a case to a class (category or label) on the basis of a known set of features or characteristics. This is usually done by a classifier learned from a training dataset. From the validation process of the classifier with a testing dataset, we obtain a confusion matrix *C*, which takes into account actual and predicted classes of the cases in the testing dataset. To fix ideas, assume that there are *N* different classes labeled {1, …, *N*}. Then, *C* = (*C*_*ij*_)_*i*,*j*=1,…,*N*_ is a *N* × *N* matrix defined by: *C*_*ij*_ is the number of cases in the testing dataset that belong to class *i* and have been assigned to class *j* by the classifier. Note that *C*_*ij*_ ≥ 0. Let *S* denote the sum of all the elements of *C* (the number of cases in the testing dataset), that is, $S=\sum_{i,j=1}^{N}C_{ij}>0$. In the binary case *N* = 2, to abbreviate notation we preferably denote $C=(\begin{array}{cc} C_{11} & C_{12}\\ C_{21} & C_{22} \end{array})$ by $\left(\begin{array}{cc} a & b\\ c & d \end{array}\right)$, as previously mentioned in the Introduction.

In the context of classification, *Accuracy* (Acc for brief) is the fraction of correctly classified cases in the testing dataset, that is, $\text{Acc}=\sum_{i=1}^{N}C_{ii}/S$. This performance measure is one of the most intuitive, and it is naturally extended to multi-class from binary classification. Acc mainly considers the diagonal of the confusion matrix, and does not take into account how the off-diagonal elements, corresponding to misclassification, are distributed.

Other more subtle performance measures based on the confusion matrix have been introduced to compare classifiers. We here compare two of the most commonly used. Note that these measures are invariant for equivalent confusion matrices.

### Matthews correlation coefficient

#### The binary case

*Matthews Correlation Coefficient* MCC was first introduced in the binary case by B.W. Matthews [4] to assess the performance of protein secondary structure prediction, as the *ϕ*-coefficient, which is the measure of association obtained by discretization of the Pearson’s correlation coefficient for two binary vectors. That is, in the binary case, MCC = *ϕ* = *ρ*(*x*, *y*), where *x* = (*x*_1_, …, *x*_*S*_)^*T*^ and *y* = (*y*_1_, …, *y*_*S*_)^*T*^ are the *S*-dimensional binary vectors defined in this way:

$$\begin{array}{cc} x_{i} & =\{\begin{array}{cc} 1 & \text{if}\;\text{case}\;i\;\text{belongs}\;\text{to}\;\text{class}\;“\text{positive}”,\\ 0 & \text{if}\;\text{it}\;\text{belongs}\;\text{to}\;\text{class}\;“\text{negative}”, \end{array}\\ y_{i} & =\{\begin{array}{cc} 1 & \text{if}\;\text{case}\;i\;\text{has}\;\text{been}\;\text{classified}\;\text{as}\;\text{belonging}\;\text{to}\;\text{class}\;“\text{positive}”,\\ 0 & \text{if}\;\text{it}\;\text{has}\;\text{been}\;\text{classified}\;\text{as}\;\text{belonging}\;\text{to}\;\text{class}\;“\text{negative}”, \end{array} \end{array}$$

and *ρ* is Pearson’s correlation coefficient defined by

$$\rho (x,y)=\frac{Cov(x,\;y)}{\sqrt{Cov(x,\;x)}\;\sqrt{Cov(y,\;y)}}=\frac{\sum_{i=1}^{S}\left(x_{i}-\bar{x}\right)\;\left(y_{i}-\bar{y}\right)}{\sqrt{\sum_{i=1}^{S}{\left(x_{i}-\bar{x}\right)}^{2}}\;\;\sqrt{\sum_{i=1}^{S}{\left(y_{i}-\bar{y}\right)}^{2}}}$$ (1)

where, as usual, $\bar{x}=\frac{1}{S}\;\sum_{i=1}^{S}x_{i}$ and $\bar{y}=\frac{1}{S}\;\sum_{i=1}^{S}y_{i}$, and *Cov*(*x*, *y*) denotes the statistical covariance of *x* and *y*, that is, $Cov\left(x,\;y\right)=\frac{1}{S}\;\sum_{i=1}^{S}\left(x_{i}-\bar{x}\right)\;\left(y_{i}-\bar{y}\right)$, and when *x* = *y*, *Cov*(*x*, *x*) = *Var*(*x*) is the statistical (uncorrected) variance of *x*. Note that the square of the *ϕ*-coefficient is related to the chi-squared statistic for the 2 × 2 contingency table, *χ*^2^, by means of $\phi^{2}=\frac{\chi^{2}}{S}$. Then, using some algebra and taking into account that, by definition of vectors *x* and *y*, the elements of the confusion matrix are

$$\begin{array}{c} a=\sum_{i=1}^{S}x_{i}\;y_{i},\;b=\sum_{i=1}^{S}x_{i}\;\left(1-y_{i}\right),\;c=\sum_{i=1}^{S}\left(1-x_{i}\right)\;y_{i}\;\;\text{and}\;\;d=\sum_{i=1}^{S}\left(1-x_{i}\right)\;\left(1-y_{i}\right), \end{array}$$

we obtain that

$$\begin{array}{cc} a\;d-b\;c & =S\;\sum_{i=1}^{S}x_{i}\;y_{i}-\left(\sum_{j=1}^{S}x_{j}\right)\;\left(\sum_{k=1}^{S}y_{k}\right),\\ a+b & =\sum_{i=1}^{S}x_{i},\;b+d=S-\sum_{i=1}^{S}y_{i},\;a+c=\sum_{i=1}^{S}y_{i},\;c+d=S-\sum_{i=1}^{S}x_{i} \end{array}$$

and then using $x_{i}^{2}=x_{i}$ and $y_{i}^{2}=y_{i}$ for any *i* = 1, …, *S*, we can rewrite (1) as

$$\text{MCC}=\frac{a\;d-b\;c}{\sqrt{\left(a+b)\;(b+d)\;(a+c)\;(c+d\right)}}\;\text{(in}\;\text{the}\;\text{binary}\;\text{case)}.$$ (2)

#### The multi-class case

In [5] the problem of evaluation of prediction of RNA secondary structure in cases where some predicted pairs go into the category of “unknown” due to lack of reliability, is considered. By introducing an extended correlation coefficient that applies to any number of categories, the author facilitates addressing the problem of predicting base pairs of RNA secondary structure as a three-category problem instead of artificially force it to fall into the binary case by fixing one of the categories, and then considering which cases belong and which do not belong to that category, leading to a loss of information and a suboptimal procedure. Indeed, MCC is generalized in [5] to classification with *N* > 2 classes based on considering the expected covariance of all categories and constructing the following extension of Pearson’s correlation coefficient *ρ* from a pair of binary vectors to a pair of binary matrices:

$$\tilde{\rho }\left(X,Y\right)=\frac{\tilde{Cov}\left(X,\;Y\right)}{\sqrt{\tilde{Cov}\left(X,\;X\right)}\;\sqrt{\tilde{Cov}\left(Y,\;Y\right)}}\;,$$ (3)

where if *X* and *Y* are two matrices *S* × *N*, $\tilde{Cov}\left(X,\;Y\right)$ is defined as the average of the *N* covariances between the different pairs of *S*-dimensional binary vectors given by the same column in matrices *X* and *Y*, that is, $\tilde{Cov}\left(X,\;Y\right)=\frac{1}{N}\;\sum_{k=1}^{N}Cov\left(x^{k},\;y^{k}\right)$, where *x*^*k*^ = (*X*_1*k*_, …, *X*_*Sk*_)^*T*^ and *y*^*k*^ = (*Y*_1*k*_, …, *Y*_*Sk*_)^*T*^ are the columns *k* of matrices *X* and *Y*, respectively. Therefore, by defining *S* × *N* matrices *X* = (*X*_*ij*_)_*i*,*j*_ and *Y* = (*Y*_*ij*_)_*i*,*j*_ in the following way:

$$\begin{array}{cc} X_{ij} & =\{\begin{array}{cc} 1 & \text{if}\;\text{case}\;i\;\text{belongs}\;\text{to}\;\text{class}\;j,\\ 0 & \text{if}\;\text{it}\;\text{belongs}\;\text{to}\;\text{other}\;\text{class}, \end{array}\\ Y_{ij} & =\{\begin{array}{cc} 1 & \text{if}\;\text{case}\;i\;\text{has}\;\text{been}\;\text{classified}\;\text{as}\;\text{belonging}\;\text{to}\;\text{class}\;j,\\ 0 & \text{if}\;\text{it}\;\text{has}\;\text{been}\;\text{classified}\;\text{as}\;\text{belonging}\;\text{to}\;\text{other}\;\text{class}, \end{array} \end{array}$$

for *i* = 1, …, *S* and *j* = 1, …, *N*, we finally introduce the multi-class extension by $\text{MCC}=\tilde{\rho }\left(X,\;Y\right)$, and by using some algebra and that by definition of matrices *X* and *Y*, $C_{ij}=\sum_{\ell =1}^{S}X_{\ell i}\;Y_{\ell j}$, we obtain the known expression

$$\text{MCC}=\frac{\sum_{k,\ell ,m=1}^{N}\left(C_{kk}\;C_{\ell m}-C_{mk}\;C_{k\ell }\right)}{\sqrt{\sum_{k=1}^{N}((\sum_{\ell =1}^{N}C_{k\ell })(\sum_{u,v=1,\;u\neq k}^{N}C_{uv}))}\;\sqrt{\sum_{k=1}^{N}((\sum_{\ell =1}^{N}C_{\ell k})(\sum_{u,v=1,\;u\neq k}^{N}C_{vu}))}}$$ (4)

We give below a sketch of the proof of the equivalence between (3) and (4). Indeed, the numerator of (3) can be developed as follows:

$$\begin{array}{cc} \tilde{Cov}\left(X,\;Y\right) & =\frac{1}{N}\;\sum_{k=1}^{N}(\frac{1}{S}\sum_{r=1}^{S}(X_{rk}-\bar{x^{k}})\;(Y_{rk}-\bar{y^{k}}))=\frac{1}{N\;S}\;\sum_{k=1}^{N}(\sum_{r=1}^{S}X_{rk}\;Y_{rk}-S\;\bar{x^{k}}\;\bar{y^{k}})\\ & =\frac{1}{N\;S^{2}}\;\sum_{k,\ell ,m=1}^{N}(C_{kk}\;C_{\ell m}-C_{k\ell }\;C_{mk}) \end{array}$$

using that $S\;\bar{x^{k}}\;\bar{y^{k}}=\frac{1}{S}\;\sum_{\ell ,m=1}^{N}C_{k\ell }\;C_{mk}$, which is a consequence of the fact that by definition, $\bar{x^{k}}=\frac{1}{S}\sum_{r=1}^{S}X_{rk}=\frac{1}{S}\sum_{\ell =1}^{N}C_{k\ell }$ since

$$\begin{array}{c} \sum_{\ell =1}^{N}C_{k\ell }=\sum_{\ell =1}^{N}(\sum_{r=1}^{S}X_{rk}\;Y_{r\ell })=\sum_{r=1}^{S}X_{rk}\;(\sum_{\ell =1}^{N}Y_{r\ell })=\sum_{r=1}^{S}X_{rk} \end{array}$$

(note that by definition of *Y*, $\sum_{\ell =1}^{N}Y_{r\ell }=1$), and analogously with $\bar{y^{k}}=\frac{1}{S}\sum_{r=1}^{S}Y_{rk}=\frac{1}{S}\sum_{m=1}^{N}C_{mk}$.

We also used that $\sum_{r=1}^{S}X_{rk}\;Y_{rk}=C_{kk}$, and that $S=\sum_{\ell ,m=1}^{N}C_{\ell m}$. Now we develop the term in the denominator of (3) corresponding to *X* (analogous development would be obtained for *Y*):

$$\begin{array}{cc} \tilde{Cov}\left(X,\;X\right) & =\frac{1}{N}\;\sum_{k=1}^{N}(\frac{1}{S}\sum_{r=1}^{S}(X_{rk}-\bar{x^{k}}{)}^{2})=\frac{1}{N\;S}\;\sum_{k=1}^{N}(\sum_{r=1}^{S}X_{rk}^{2}-S\;{\left(\bar{x^{k}}\right)}^{2})\\ & =\frac{1}{N\;S}\;\sum_{k=1}^{N}(\sum_{\ell =1}^{N}C_{k\ell }-\frac{1}{S}\;(\sum_{v=1}^{N}C_{kv}{)}^{2})\;\text{where}\;\text{we}\;\text{use}\;\text{that}\;X_{rk}^{2}=X_{rk}\\ & =\frac{1}{N\;S^{2}}\;\sum_{k=1}^{N}((\sum_{\ell =1}^{N}C_{k\ell })\;(S-\sum_{v=1}^{N}C_{kv}))\\ & =\frac{1}{N\;S^{2}}\;\sum_{k=1}^{N}((\sum_{\ell =1}^{N}C_{k\ell })\;(\sum_{u,v=1,u\neq k}^{N}C_{uv}))\;\text{using}\;\text{that}\;S=\sum_{u,v=1}^{N}C_{uv}. \end{array}$$

Note that in the binary case, expression (4) matches (2). Indeed, when *N* = 2, numerator of (4) can be written as 2(*C*_11_ *C*_22_ − *C*_21_ *C*_12_) = 2(*ad* − *bc*), while the first term in the denominator is $\sqrt{2\;(a+b)\;(c+d)}$, and the second one coincides with $\sqrt{2\;(a+c)\;(b+d)}$.

Software provided by the author of [5] allowing to perform the calculations easily is available at http://rk.kvl.dk/.

### Cohen’s *Kappa*

Cohen’s *Kappa* statistic, or simply *Kappa* (henceforth, also denoted by K), was originally introduced by J. A. Cohen [27] in the field of psychology as a measure of agreement between two judge, and later it has been used in the literature as a performance measure in classification, as for example in [46]. More concretely, *Kappa* is used in classification as a measure of agreement between observed and predicted or inferred classes for cases in a testing dataset. Its definition is:

$$K=\frac{\text{Acc}-P_{e}}{1-P_{e}}\;,$$ (5)

where *P*_*e*_ is the hypothetical probability of chance agreement, using the values of the confusion matrix to estimate the probabilities of randomly choose each class, that is, $P_{e}=\sum_{i=1}^{N}\frac{C_{i\;\cdot }\times C_{\cdot \;i}}{S^{2}}$, where as usual, we use the notations $C_{i\;\cdot }=\sum_{j=1}^{N}C_{ij}$ (the sum of row *i*), and $C_{\cdot \;j}=\sum_{\ell =1}^{N}C_{\ell j}$ (the sum of column *j*).

Both MCC and *Kappa* assume their theoretical maximum value of +1 when classification is perfect, the larger the metric value, the better the classifier performance. MCC ranges between −1 and +1 while *Kappa* does not in general, although it does in the cases considered in this work. Moreover, it is straightforward to see that they are symmetric, that is, $K\left(C^{T}\right)=K\left(C\right)$ and MCC(*C*^*T*^) = MCC(*C*).

## The symmetric case

In the case of a symmetric confusion matrix, it is known that *Kappa* statistic is equivalent to *Scott’s pi* ([28], [47]), which is a special case of *Krippendorff’s alpha* ([48]). *Scott’s pi* is a statistic with the same structure as *Kappa* but that differs from it in the definition of *P*_*e*_. Hereunder, we will show that if *C* is a symmetric matrix, *Kappa* and MCC not only are consistent with each other but they coincide exactly. Although this result seems to be known, we could not find a reference for it and therefore, we provide its proof here.

**Proposition 1** *Let C* = (*C*_*ij*_)_*i*,*j*=1,…,*N*_ *be a symmetric confusion matrix in the general multi-class setting. That is, C* = *C*^*T*^. *Then*, K(C)=MCC(C).

*Proof*. By (4) and taking into account that *C*_*ij*_ = *C*_*ji*_ by symmetry, we can write

$$\begin{array}{cc} \text{MCC}(C) & =\frac{\sum_{k,\ell ,m=1}^{N}\left(C_{kk}C_{\ell m}-C_{mk}C_{k\ell }\right)}{\sum_{k=1}^{N}(\left(\sum_{\ell =1}^{N}C_{k\ell }\right)\left(\sum_{u,v=1,u\neq k}^{N}C_{uv}\right))}=\frac{\sum_{k=1}^{N}C_{kk}\left(\sum_{\ell ,m=1}^{N}C_{\ell m}\right)-\sum_{k,\ell ,m=1}^{N}C_{km}C_{k\ell }}{\sum_{k=1}^{N}(\left(\sum_{\ell =1}^{N}C_{k\ell }\right)\left(S-\sum_{v=1}^{N}C_{kv}\right))}\\ & =\frac{S\;\sum_{k=1}^{N}C_{kk}-\sum_{k=1}^{N}{\left(\sum_{\ell =1}^{N}C_{k\ell }\right)}^{2}}{S\;\sum_{k,\ell =1}^{N}C_{k\ell }-\sum_{k=1}^{N}{\left(\sum_{\ell =1}^{N}C_{k\ell }\right)}^{2}}=\frac{S\;\sum_{k=1}^{N}C_{kk}-\sum_{k=1}^{N}{\left(\sum_{\ell =1}^{N}C_{k\ell }\right)}^{2}}{S^{2}-\sum_{k=1}^{N}{\left(\sum_{\ell =1}^{N}C_{k\ell }\right)}^{2}}\;. \end{array}$$ (6)

On the other hand, by symmetry we can write $P_{e}=\sum_{k=1}^{N}C_{k\;\cdot }^{2}/S^{2}$, and therefore,

$$\begin{array}{c} K\left(C\right)=\frac{\frac{\sum_{k=1}^{N}C_{kk}}{S}-\frac{\sum_{k=1}^{N}{\left(\sum_{\ell =1}^{N}C_{k\ell }\right)}^{2}}{S^{2}}}{1-\frac{\sum_{k=1}^{N}{\left(\sum_{\ell =1}^{N}C_{k\ell }\right)}^{2}}{S^{2}}}=\frac{S\;\sum_{k=1}^{N}C_{kk}-\sum_{k=1}^{N}{\left(\sum_{\ell =1}^{N}C_{k\ell }\right)}^{2}}{S^{2}-\sum_{k=1}^{N}{\left(\sum_{\ell =1}^{N}C_{k\ell }\right)}^{2}}, \end{array}$$

which coincides with MCC(*C*) by (6).

## The binary case

Let *C* be a generic confusion matrix in dimension 2, $C=(\begin{array}{cc} a & b\\ c & d \end{array})$. By (2) and (5), we have that

$$\begin{array}{cc} \text{MCC}(C) & =\frac{ad-bc}{\sqrt{\left(a+b)\;(b+d)\;(a+c)\;(c+d\right)}}\;\text{and}\\ K(C) & =\frac{2\;(ad-bc)}{\left(a+b)\;(b+d)+(a+c)\;(c+d\right)} \end{array}$$

and it turns out that K(C) is the harmonic mean of *α* and *β*, while MCC(*C*) is their geometric mean, being

$$\begin{array}{c} \alpha =\frac{ad-bc}{\left(a+b)\;(b+d\right)}\;\text{and}\;\beta =\frac{ad-bc}{\left(a+c)\;(c+d\right)}\;. \end{array}$$

That is, $K\left(C\right)=\frac{2}{\frac{1}{\alpha }+\frac{1}{\beta }}$ and $\text{MCC}\left(C\right)=\sqrt{\alpha \;\beta }$. As a direct consequence of the known relationship between these two means, we have that in the binary case:

$$\begin{array}{cc} & \min(\alpha ,\;\beta )\leq K(C),\;\;\text{MCC}(C)\leq \max(\alpha ,\;\beta )\;\text{and}\\ & \left\{ \begin{array}{cc} & \text{If}\;ad>bc,\;0<K(C)\leq \text{MCC}(C)\text{,}\\ & \text{If}\;ad<bc,\;\text{MCC}(C)\leq K(C)<0\text{,}\\ & \text{If}\;ad=bc,\;\text{MCC}(C)=K(C)=0\text{.} \end{array}\right. \end{array}$$ (7)

Now we delve a little deeper into the relationship between the two performance measures. By the property of invariance for equivalent confusion matrices, we can split the study of the binary case into two different scenarios: *c* = 0 and *c* = 1 (the latter corresponding to *c* > 0). These two cases cover all the possibilities, determining a partition of the set of binary confusion matrices into two subsets with clearly differentiated behaviour. As we will see next, when *c* = 0 there is an agreement between MCC and *Kappa*. What is more, MCC and *Kappa* are linked by means of a functional relationship (see Proposition 2 below) that easily shows the relationship of monotony between them, which implies that when one of them grows or decreases, the other also does the same, that is, they have a consistent behaviour. On the contrary, when *c* = 1 an important disagreement between the two measures highlights in different particular scenarios (see Corollaries 4, 5 and 6). Indeed, in all of them it is shown that while MCC monotonically decreases as the task done by the classifier is getting worse, *Kappa* does not.

Moreover, as the row sums are the actual number of cases in the testing dataset belonging to each class, we assume that they are both strictly positive, that is, *a* + *b* > 0 and *c* + *d* > 0. We also must ensure that MCC can be calculated, i.e, that we do not divide by zero. For that, the sum of the columns must also be strictly positive, that is, we additionally assume that *a* + *c* > 0 and *b* + *d* > 0.

### *The c* = 0 *case: Agreement between* MCC *and Kappa*

This case corresponds to perfect classification of the negative class, since there are no cases of the negative class in the testing dataset that have been classified as belonging to the positive class. Then, we assume *a* > 0 and *d* > 0. Moreover, we assume *b* > 0 since *b* = 0 corresponds to the symmetric case already studied in the previous section, in which K=MCC=1. We use notation $C_{0}=(\begin{array}{cc} a & b\\ 0 & d \end{array}).$ We have, then,

$$\begin{array}{cc} \text{MCC}(C_{0}) & =\sqrt{\frac{a\;d}{\left(a+b)(b+d\right)}}\;\text{and}\;K\left(C_{0}\right)=\frac{2\;a\;d}{a\;d+(a+b)\;(b+d)}\;. \end{array}$$

We will show that in this case there is agreement between the behaviour of the two measures. Indeed, they are linked by means of a functional relationship, as can be seen in the next proposition.

**Proposition 2**

$$\begin{array}{c} K\left(C_{0}\right)=\frac{2\;{\left(\text{MCC}\left(C_{0}\right)\right)}^{2}}{1+{\left(\text{MCC}\left(C_{0}\right)\right)}^{2}}\;, \end{array}$$

*and the following properties hold*:

1. *Since* MCC(*C*_0_) > 0, $K(C_{0})$ *is a monotonically increasing function of* MCC(*C*_0_), *so they are consistent performance measures*.
2. $0<\frac{ad}{\left(a+b)\;(b+d\right)}<K\left(C_{0}\right)<\text{MCC}\left(C_{0}\right)<1$.
3. *The maximum distance between them is achieved when* MCC(*C*_0_) ≈ 0.3, *and is* ≈ 0.13.

**Fig 1:**
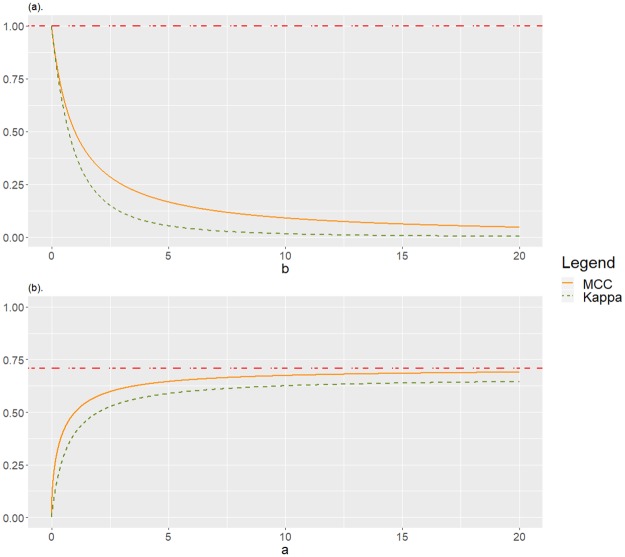
Agreement between MCC and *Kappa* for *C*_0_. Unbalanced case with underrepresentation of the negative class, which is perfectly classified. (a) With *a* = *d* = 1, as function of *b*: positive class mainly misclassified. (b) With *b* = *d* = 1 as function of *a*: positive class mainly well classified.

Moreover,

- Fixed *a*, *d*,
  $$\begin{array}{c} \underset{b\to +\infty }{\text{lim}}\text{MCC}\left(C_{0}\right)=\underset{b\to +\infty }{\text{lim}}K\left(C_{0}\right)=0\;, \end{array}$$
  which corresponds to an scenario in which the negative class is underrepresented and cases actually in the positive class are mainly misclassified. On the other hand,
  $$\begin{array}{c} \underset{b\to 0}{\text{lim}}\text{MCC}\left(C_{0}\right)=\underset{b\to 0}{\text{lim}}K\left(C_{0}\right)=1\;, \end{array}$$
  corresponding to perfect classification (see Fig 1(a)).
- Fixed *b*, *d*,
  $$\begin{array}{c} 0<\underset{a\to +\infty }{\text{lim}}K\left(C_{0}\right)=\frac{2\;d}{2\;d+b}<\underset{a\to +\infty }{\text{lim}}\text{MCC}\left(C_{0}\right)=\sqrt{\frac{d}{b+d}}<1\;, \end{array}$$
  which corresponds to an scenario in which the negative class is underrepresented but cases actually in the positive class are mainly well classified. Note that as *b* → 0, both ${\text{lim}}_{a\to +\infty }K\left(C_{0}\right)$ and lim_a→+∞_ MCC(*C*_0_), tend to be 1.
  On the other hand,
  $$\begin{array}{c} \underset{a\to 0}{\text{lim}}\text{MCC}\left(C_{0}\right)=\underset{a\to 0}{\text{lim}}K\left(C_{0}\right)=0\;, \end{array}$$
  corresponding to complete misclassification of the positive class (see Fig 1(b)).
- The case with *a*, *b* fixed, considering MCC(*C*_0_) and $K(C_{0})$ as function of *d*, is symmetric to the previous one, and then omitted.

### *The c* = 1 *case: Disagreement between* MCC *and Kappa*

This case corresponds to not-completely perfect classification of the negative class, since there is at least one case in the testing dataset belonging to this class that has been classified as being in the positive class. We assume *b* > 0 since if *b* = 0 we are in the previous situation, by symmetry of MCC and *Kappa*. Although *b* = 1 corresponds to a symmetric confusion matrix already studied, we include it in this section for the sake of completeness. We use the notation $C_{1}=(\begin{array}{cc} a & b\\ 1 & d \end{array}).$ Then,

$$\begin{array}{cc} \text{MCC}(C_{1}) & =\frac{a\;d-b}{\sqrt{\left(a+1)\;(a+b)\;(d+1)\;(d+b\right)}}\;,\\ K(C_{1}) & =\frac{2\;(a\;d-b)}{\left(a+1)\;(d+1)+(a+b)\;(d+b\right)} \end{array}$$

**Proposition 3** *If* $a=d=0,\;b\neq 1,\;-1=\text{MCC}\left(C_{1}\right)<K\left(C_{1}\right)=\frac{-2\;b}{1+b^{2}}<0\;$.

*If* $a=d=0,\;b=1,\;\text{MCC}\left(C_{1}\right)=K\left(C_{1}\right)=-1\;$.

*Otherwise*,

$$\begin{array}{c} \left\{ \begin{array}{cc} if\;\;ad=b, & \text{MCC}\left(C_{1}\right)=K\left(C_{1}\right)=0\;,\\ if\;\;ad>b, & \left\{ \begin{array}{cc} if\;b>1, & 0<\frac{ad-b}{\left(a+b)(d+b\right)}<K\left(C_{1}\right)<\text{MCC}\left(C_{1}\right)<\frac{ad-b}{\left(a+1)(d+1\right)}<1,\\ if\;b=1, & 0<K\left(C_{1}\right)=\text{MCC}\left(C_{1}\right)=\frac{ad-1}{\left(a+1)(d+1\right)}<1,\\ if\;b<1, & 0<\frac{ad-b}{\left(a+1)(d+1\right)}<K\left(C_{1}\right)<\text{MCC}\left(C_{1}\right)<\frac{ad-b}{\left(a+b)(d+b\right)}<1, \end{array}\right.\\ if\;\;ad<b, & \left\{ \begin{array}{cc} if\;b>1, & \text{max}\;\left(-1,\frac{ad-b}{\left(a+1)(d+1\right)}\right)<\text{MCC}\left(C_{1}\right)<K\left(C_{1}\right)<\frac{ad-b}{\left(a+b)(d+b\right)}<0,\\ if\;b=1, & -1<\text{MCC}\left(C_{1}\right)=K\left(C_{1}\right)=\frac{ad-1}{\left(a+1)(d+1\right)}<0,\\ if\;b<1, & \text{max}\;\left(-1,\frac{ad-b}{\left(a+b)(d+b\right)}\right)<\text{MCC}\left(C_{1}\right)<K\left(C_{1}\right)<\frac{ad-b}{\left(a+1)(d+1\right)}<0. \end{array}\right. \end{array}\right. \end{array}$$

Next we consider some particular scenarios of this case that should be explored.

**Fig 2:**
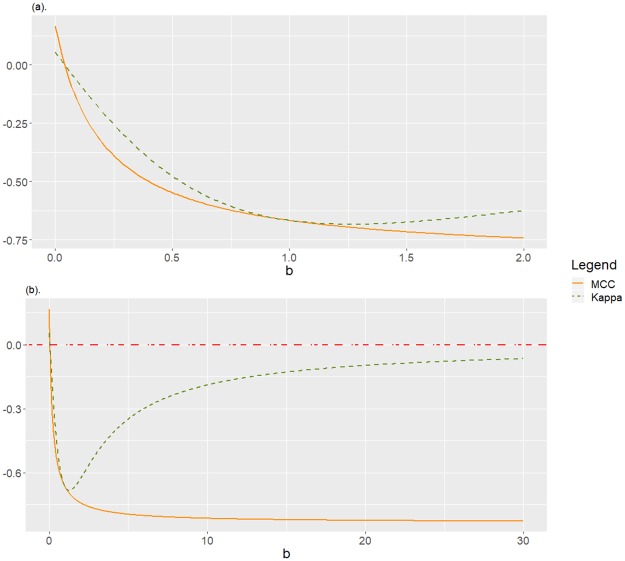
Disagreement between MCC and *Kappa* for $C_{1,a}^{a,b}$ with *a* = 0.2, as function of *b* ≥ 0. If *b* > 1, the negative class is underrepresented and quite misclassified, and the positive class is mainly misclassified. (a) A zoom of the detail for *b* ≤ 2. (b) For *b* ≤ 30.

**Fig 3:**
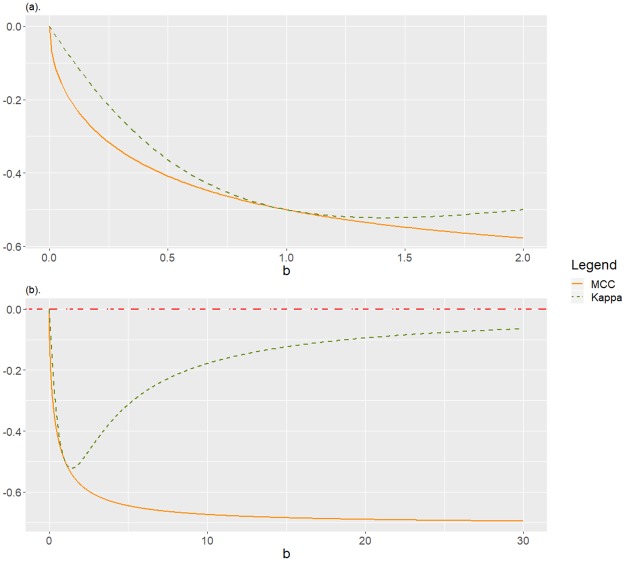
Disagreement between MCC and *Kappa* for $C_{1,0}^{a,b}$ with *a* = 1, as function of *b* ≥ 0. If *b* > 1, the negative class is underrepresented and systematically misclassified, and the positive class is also mainly misclassified. (a) A zoom of the detail for *b* ≤ 2. (b) For *b* ≤ 30.

**Fig 4:**
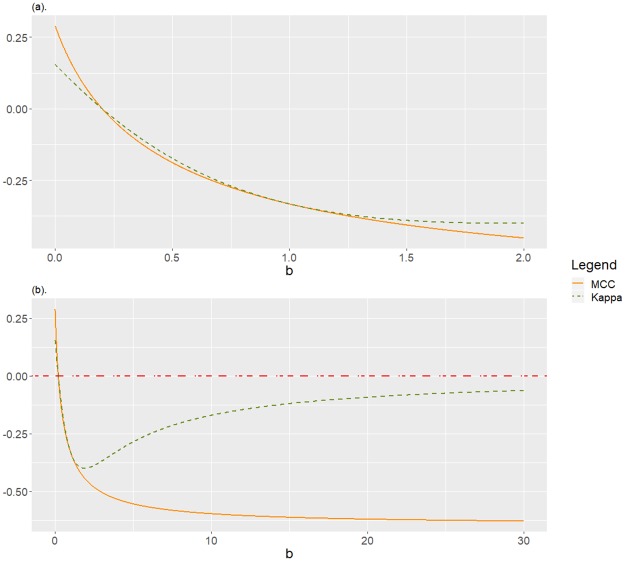
Disagreement between MCC and *Kappa* for $C_{1,1}^{a,b}$ with *a* = 0.2, as function of *b* ≥ 0. The negative class is classified at random. If *b* > 1 the positive class is mainly misclassified, and the negative class is underrepresented. (a) A zoom of the detail for *b* ≤ 2. (b) For *b* ≤ 30.

1. *a* = *d* > 0.
  We use notation $C_{1,a}^{a,b}=(\begin{array}{cc} a & b\\ 1 & a \end{array})$. Fixed *a* > 0, if *b* > 1, the negative class is underrepresented, and the positive class is mainly misclassified, while if *b* < 1, say *b* = 1/*h* with *h* > 1, $C_{1,a}^{a,b}\equiv (\begin{array}{cc} h\;a & 1\\ h & h\;a \end{array})$, which is a confusion matrix that corresponds to underrepresentation of the positive class while it is mainly well classified (if *b* → 0, which is equivalent to *h* → +∞). Then,
  $$\begin{array}{cc} \text{MCC}(C_{1,a}^{a,b}) & =\frac{a^{2}-b}{\left(a+1)\;(a+b\right)}\;\text{and}\;K\left(C_{1,a}^{a,b}\right)=\frac{2\;(a^{2}-b)}{{\left(a+1\right)}^{2}+{\left(a+b\right)}^{2}}\;. \end{array}$$
  From these expressions and Proposition 3, we obtain:
  **Corollary 4** *If* $a=d=0,\;b\neq 1,\;-1=\text{MCC}\left(C_{1}\right)<K\left(C_{1}\right)=\frac{-2\;b}{1+b^{2}}<0\;$.
  *If* $a=d=0,\;b=1,\;\text{MCC}\left(C_{1}\right)=K\left(C_{1}\right)=-1\;$.
  *Otherwise*,
  $$\begin{array}{c} \left\{ \begin{array}{cc} if\;\;a^{2}=b, & \text{MCC}\left(C_{1}\right)=K\left(C_{1}\right)=0\;,\\ if\;\;a^{2}>b, & \left\{ \begin{array}{cc} if\;b>1, & 0<\frac{a^{2}-b}{{\left(a+b\right)}^{2}}<K\left(C_{1}\right)<\text{MCC}\left(C_{1}\right)<\frac{a^{2}-b}{{\left(a+1\right)}^{2}}<1,\\ if\;b=1, & 0<K\left(C_{1}\right)=\text{MCC}\left(C_{1}\right)=\frac{a-1}{\left(a+1\right)}<1,\\ if\;b<1, & 0<\frac{a^{2}-b}{{\left(a+1\right)}^{2}}<K\left(C_{1}\right)<\text{MCC}\left(C_{1}\right)<\frac{a^{2}-b}{{\left(a+b\right)}^{2}}<1, \end{array}\right.\\ if\;\;a^{2}<b, & \left\{ \begin{array}{cc} if\;1<b<{\left(a+1\right)}^{2}+a^{2}, & \\ -1<\frac{a^{2}-b}{{\left(a+1\right)}^{2}}<\text{MCC}\left(C_{1}\right)<K\left(C_{1}\right)<\frac{a^{2}-b}{{\left(a+b\right)}^{2}}<0, & \\ if\;{\left(a+1\right)}^{2}+a^{2}\leq b,-1<\text{MCC}\left(C_{1}\right)<K\left(C_{1}\right)<\frac{a^{2}-b}{{\left(a+b\right)}^{2}}<0, & \\ if\;b=1,-1<\text{MCC}\left(C_{1}\right)=K\left(C_{1}\right)=\frac{a-1}{\left(a+1\right)}<0, & \\ if\;(b=1-\frac{\sqrt{2}}{2}\;\text{and}\;a=\frac{\sqrt{2}-1}{2})\;\text{or}\;(b\in \left\{b_{1},\;b_{2}\right\}\;\text{and}\;0<a<\frac{\sqrt{2}-1}{2}), & \\ -1=\frac{a^{2}-b}{{\left(a+b\right)}^{2}}<\text{MCC}\left(C_{1}\right)<K\left(C_{1}\right)<\frac{a^{2}-b}{{\left(a+1\right)}^{2}}<0, & \\ if\;b_{1}<b<b_{2}\;\text{and}\;0<a<\frac{\sqrt{2}-1}{2}, & \\ -1<\text{MCC}\left(C_{1}\right)<K\left(C_{1}\right)<\frac{a^{2}-b}{{\left(a+1\right)}^{2}}<0, & \\ Otherwise, & \\ -1<\frac{a^{2}-b}{{\left(a+b\right)}^{2}}<\text{MCC}\left(C_{1}\right)<K\left(C_{1}\right)<\frac{a^{2}-b}{{\left(a+1\right)}^{2}}<0, \end{array}\right. \end{array}\right. \end{array}$$
  *where*
  $$\begin{array}{c} 0<b_{1}=\frac{\left(1-2\;a\right)-\sqrt{{\left(1-2\;a\right)}^{2}-8\;a^{2}}}{2}<b_{2}=\frac{\left(1-2\;a\right)+\sqrt{{\left(1-2\;a\right)}^{2}-8\;a^{2}}}{2}<1\;. \end{array}$$
  *Fixed a* > 0, $-1<\lim_{b\to +\infty }\text{MCC}\left(C_{1,a}^{a,b}\right)=-\frac{1}{a+1}<\lim_{b\to +\infty }K\left(C_{1,a}^{a,b}\right)=0\;$, *while*
  $$\begin{array}{c} 0<\frac{a^{2}}{{\left(a+1\right)}^{2}}<\underset{b\to 0}{\text{lim}}K\left(C_{1,a}^{a,b}\right)=\frac{2\;a^{2}}{{\left(a+1\right)}^{2}+a^{2}}<\underset{b\to 0}{\text{lim}}\text{MCC}\left(C_{1,a}^{a,b}\right)=\frac{a}{a+1}<1 \end{array}$$
  *and* $\text{MCC}(C_{1,a}^{a,b})$, *as a function of b, is monotonically decreasing when b increases, which agrees with the intuition, since when b monotonically increases, the task done by the classifier is clearly getting worse, while* $K(C_{1,a}^{a,b})$ *is not*. *Indeed*, *fixed a* > 0, $K(C_{1,a}^{a,b})$ *has a global minimum at b* = *b*_0_ *with*
  $$b_{0}=a^{2}+\left(a+1\right)\;\sqrt{a^{2}+1}>a^{2}\;.$$
  See Fig 2 to observe the behaviour of MCC and *Kappa* fixed *a* = 0.2, as function of *b*.
  **Remark 1** *Corollary 4 explains the behaviour of* MCC *and Kappa for a confusion matrix equivalent to* $C_{1,a}^{a,b}=(\begin{array}{cc} a & b\\ 1 & a \end{array})$, *according to the values of a = “true positive” = “true negative”, and b = “false negative”/“false positive”. In particular, fixed “true positive” = “true negative” and “false positive”, we observe a contradictory behaviour between these two performance measures as b increases. Indeed, as “false negative”/“false positive” is increasing (implying that the negative class is underrepresented, and the positive class is mainly misclassified)*, MCC *monotonically decreases, what is reasonable, but Kappa does not. In fact, Kappa decreases for low values of b* (*b* < *b*_0_) *but increases otherwise. This unreasonable behaviour of Kappa goes in the direction of the thesis defended in this work*. Fig 2 *graphically shows this fact for the particular case a* = 0.2, *corresponding to a confusion matrix equivalent to* $(\begin{array}{cc} 1 & 5\;b\\ 5 & 1 \end{array})\;$.
  Case *b* > 1, with *a* = 1, corresponds to matrix *Z*_*A*_ with *A* = *b* and dimension *N* = 2, which is a pathological situation that will be studied in the next section.
2. *a* > 0, *d* = 0.
  We use notation $C_{1,0}^{a,b}=(\begin{array}{cc} a & b\\ 1 & 0 \end{array})$. In this case,
  $$\text{MCC}(C_{1,0}^{a,b})=-\sqrt{\frac{b}{\left(a+1)\;(a+b\right)}}\;\text{and}\;K\left(C_{1,0}^{a,b}\right)=-\frac{2\;b}{\left(a+1)+b\;(a+b\right)}\;.$$
  and application of Proposition 3 allows obtaining the following result:
  **Corollary 5**
  $$\begin{array}{c} \left\{ \begin{array}{cc} if\;1<b<a+1, & -1<\frac{-b}{a+1}<\text{MCC}\left(C_{1}\right)<K\left(C_{1}\right)<\frac{-1}{a+b}<0,\\ if\;a+1\leq b, & -1<\text{MCC}\left(C_{1}\right)<K\left(C_{1}\right)<\frac{-1}{a+b}<0,\\ if\;b=1 & -1<\text{MCC}\left(C_{1}\right)=K\left(C_{1}\right)=\frac{-1}{a+1}<0,\\ if\;b<1<a+b, & -1<\frac{-1}{a+b}<\text{MCC}\left(C_{1}\right)<K\left(C_{1}\right)<\frac{-b}{a+1}<0,\\ if\;a+b\leq 1, & -1<\text{MCC}\left(C_{1}\right)<K\left(C_{1}\right)<\frac{-b}{a+1}<0. \end{array}\right. \end{array}$$
  *Although fixed a* > 0, $\text{MCC}(C_{1,0}^{a,b})$ *is a monotonically decreasing function of b, coinciding with intuition*, $K(C_{1,0}^{a,b})$ *is not, achieving its global minimum when* $b=\sqrt{a+1}$. *Moreover, fixed a* > 0,
  $$\begin{array}{c} -1<\underset{b\to +\infty }{\text{lim}}\text{MCC}\left(C_{1,0}^{a,b}\right)=-\frac{1}{\sqrt{a+1}}<\underset{b\to +\infty }{\text{lim}}K\left(C_{1,0}^{a,b}\right)=0\;,\\ \;\underset{b\to 0}{\text{lim}}\text{MCC}\left(C_{1,0}^{a,b}\right)=\underset{b\to 0}{\text{lim}}K\left(C_{1,0}^{a,b}\right)=0\;. \end{array}$$
  See Fig 3 to observe the behaviour of MCC and *Kappa*, fixed *a* = 1, as function of *b*.
  **Remark 2** *In Corollary 5 we can observe the behaviour of* MCC *and Kappa for a confusion matrix equivalent to* $C_{1,0}^{a,b}=(\begin{array}{cc} a & b\\ 1 & 0 \end{array})$, *corresponding to a scenario in which the negative class is underrepresented and the classifier systematically misclassifies this class, and generally also misclassifies the positive class if b = “false negative”/“false positive” is big. In particular, fixed “true positive” and “false positive”, we observe a contradictory behaviour between* MCC *and Kappa as b increases: while* MCC *monotonically decreases, what is expected, Kappa decreases for* $b<\sqrt{a+1}$ *but increases otherwise. Again, we observe here an unreasonable behaviour of Kappa, which is graphically showed in* Fig 3 *for the particular case a* = 1, *corresponding to a confusion matrix equivalent to* $(\begin{array}{cc} 1 & b\\ 1 & 0 \end{array})\;$.
3. *d* = 1, *a* ≥ 0.
  We use notation $C_{1,1}^{a,b}=(\begin{array}{cc} a & b\\ 1 & 1 \end{array}).$ Classification of negative class is entirely done by random, that is, with the same probability a case actually in the negative class is classified as belonging to any of the two classes. If *a*, *b* > 1, negative class is underrepresented. We have that
  $$\begin{array}{c} \text{MCC}\left(C_{1,1}^{a,b}\right)=\frac{a-b}{\sqrt{2\;(a+1)\;(b+1)\;(a+b)}},\;\;K\left(C_{1,a}^{a,b}\right)=\frac{2\;(a-b)}{2\;(a+1)+(b+1)\;(a+b)} \end{array}$$
  and application of Proposition 3 gives:
  **Corollary 6**
  $$\begin{array}{c} \left\{ \begin{array}{cc} if\;\;a=b, & \text{MCC}\left(C_{1,1}^{a,b}\right)=K\left(C_{1,1}^{a,b}\right)=0\;,\\ if\;\;a>b, & \left\{ \begin{array}{cc} if\;b>1, & 0<\frac{a-b}{\left(a+b)(b+1\right)}<K\left(C_{1,1}^{a,b}\right)<\text{MCC}\left(C_{1,1}^{a,b}\right)<\frac{a-b}{2(a+1)}<1,\\ if\;b=1, & 0<K\left(C_{1,1}^{a,b}\right)=\text{MCC}\left(C_{1,1}^{a,b}\right)=\frac{a-1}{2(a+1)}<1,\\ if\;b<1, & 0<\frac{a-b}{2(a+1)}<K\left(C_{1,1}^{a,b}\right)<\text{MCC}\left(C_{1,1}^{a,b}\right)<\frac{a-b}{\left(a+b)(b+1\right)}<1, \end{array}\right.\\ if\;\;a<b, & \left\{ \begin{array}{cc} if\;1<b<3a+2, & \\ -1<\frac{a-b}{2(a+1)}<\text{MCC}\left(C_{1,1}^{a,b}\right)<K\left(C_{1,1}^{a,b}\right)<\frac{a-b}{\left(a+b)(b+1\right)}<0, & \\ if\;3a+2\leq b, & \\ -1<\text{MCC}\left(C_{1,1}^{a,b}\right)<K\left(C_{1,1}^{a,b}\right)<\frac{a-b}{\left(a+b)(b+1\right)}<0, & \\ if\;b=1, & \\ -1<\text{MCC}\left(C_{1,1}^{a,b}\right)=K\left(C_{1,1}^{a,b}\right)=\frac{a-1}{2(a+1)}<0, & \\ if\;b<1, & \\ -1<\frac{a-b}{\left(a+b)(b+1\right)}<\text{MCC}\left(C_{1,1}^{a,b}\right)<K\left(C_{1,1}^{a,b}\right)<\frac{a-b}{2(a+1)}<0. \end{array}\right. \end{array}\right. \end{array}$$
  *As in the previous cases with c* = 1, *although if we fix a* > 0, *then* $\text{MCC}(C_{1,1}^{a,b})$ *is a monotonically decreasing function of b, coinciding with intuition, we can see that* $K(C_{1,1}^{a,b})$ *is not, achieving its global minimum when* $b=a+\sqrt{2}\;\left(a+1\right)$. *Moreover, fixed a* > 0,
  $$\begin{array}{cc} -1<\underset{b\to +\infty }{\text{lim}}\;\text{MCC}\left(C_{1,1}^{a,b}\right) & =-\frac{1}{\sqrt{2\;(a+1)}}<\underset{b\to +\infty }{\text{lim}}\;K\left(C_{1,1}^{a,b}\right)=0\;,\\ 0<\frac{a}{2\;(a+1)}<\underset{b\to 0}{\text{lim}}\;K\left(C_{1,1}^{a,b}\right) & =\frac{2\;a}{3\;a+2}<\underset{b\to 0}{\text{lim}}\;\text{MCC}\left(C_{1,1}^{a,b}\right)=\sqrt{\frac{a}{2\;(a+1)}}<1\;. \end{array}$$
  In Fig 4 we can observe the behaviour of MCC and *Kappa*, fixed *a* = 0.2, as function of *b*.
  **Remark 3** *Finally, Corollary 6 is dedicated to confusion matrices equivalent to* $C_{1,1}^{a,b}=(\begin{array}{cc} a & b\\ 1 & 1 \end{array})$, *which correspond to an unbalanced database set if a, b* > 1, *with minority class the negative one, which is randomly classified, that is, each class is imputed with the same probability to a case actually in the negative class. In addition, if fixed a = “true positive”/“true negative”, when b = “false negative”/“false positive” increases the positive class is mainly misclassified. While* MCC *in this situation behaves as expected and monotonically decreases, Kappa does not, increasing for* $b>a+\sqrt{2}\;\left(a+1\right)$. *As in the previous corollaries, an unreasonable behaviour of Kappa is observed, which is shown in* Fig 4 *for the particular case a* = 0.2, *that is, for a confusion matrix equivalent to* $(\begin{array}{cc} 1 & 5\;b\\ 5 & 5 \end{array})\;$.

## The *Z*_*A*_ family

Finally, we consider another situation that highlights the incoherent behaviour of *Kappa*. {*Z*_*A*_, *A* ≥ 0} has been introduced in [2] as a family of confusion matrices useful to analyse performance measures in unbalanced situations. The definition of *Z*_*A*_ is as follows: $Z_{A}=(\begin{array}{cccc} 1 & 1 & \ldots & A\\ 1 & 1 & \ldots & 1\\ \vdots & \vdots & \ddots & \vdots \\ 1 & 1 & \ldots & 1 \end{array})$. We denote by MCC(*A*) and K(A), respectively, the MCC and *Kappa* values of matrix *Z*_*A*_. Note that when *N* = 2, this family is a particular case of iii) with *a* = 1 and *b* = *A*. Then, we obtain from Corollary 6 the following result:

**Corollary 7** *If N* = 2,

$$\text{MCC}(A)=\frac{1-A}{2\;(1+A)}\;\text{and}\;K\left(A\right)=\frac{2\;(1-A)}{4+{\left(1+A\right)}^{2}}\;.$$

*We have that*

$$\begin{array}{c} \left\{ \begin{array}{cc} If\;A=1, & K(A)=\text{MCC}(A)=0\;,\\ If\;A<1, & 0\;\;\;<\frac{1-A}{4}<K\left(A\right)<\text{MCC}\left(A\right)\;\;\;<\frac{1-A}{{\left(1+A\right)}^{2}}<1\;,\\ If\;1<A<5, & -1<\frac{1-A}{4}<\;\;\;\text{MCC}\left(A\right)<K\left(A\right)\;\;\;<\frac{1-A}{{\left(1+A\right)}^{2}}<0\;,\\ If\;5\leq A, & -1<\;\;\;\text{MCC}\left(A\right)<K\left(A\right)\;\;\;<\frac{1-A}{{\left(1+A\right)}^{2}}<0\;. \end{array}\right. \end{array}$$

*Although* MCC(*A*) *is a monotonically decreasing function of A, coinciding with intuition*, K(A) *is not, achieving its global minimum when* $A=1+2\;\sqrt{2}>1$. *Moreover*,

$$\begin{array}{cc} -1<\underset{A\to +\infty }{\text{lim}}\;\text{MCC}\left(A\right) & =-\frac{1}{2}<\underset{A\to +\infty }{\text{lim}}\;K\left(A\right)=0\;,\\ 0<\frac{1}{4}<\underset{A\to 0}{\text{lim}}\;K\left(A\right) & =\frac{2}{5}<\underset{A\to 0}{\text{lim}}\;\text{MCC}\left(A\right)=\frac{1}{2}<1\;. \end{array}$$

We generalize the previous result to any *N* ≥ 2 in the following proposition:

**Proposition 8**

$$\begin{array}{cc} \text{MCC}(A) & =\frac{1-A}{\left(N-1\right)\;(N^{2}-2\left(1-A\right))},\\ K(A) & =N\;\frac{1-A}{{\left(1-A\right)}^{2}-2\;N\;\left(N-1\right)\;\left(1-A\right)+N^{3}\;\left(N-1\right)}, \end{array}$$

*and the following properties hold*:

1. MCC(1)=K(1)=0,
2. $\frac{1}{\text{MCC}(A)}-\frac{1}{K(A)}=\frac{A-1}{N}$ *and then*,
  $$\begin{array}{c} \left\{ \begin{array}{cc} If\;A<1, & 0<K(A)<\text{MCC}(A)<1\;,\\ If\;1<A, & -1<\text{MCC}(A)<K(A)<0\;, \end{array}\right. \end{array}$$
$-1<{\text{lim}}_{A\to \infty }\text{MCC}\left(A\right)=\frac{-1}{2\;(N-1)}<{\text{lim}}_{A\to \infty }K\left(A\right)=0\;$,
4. $0<{\text{lim}}_{A\to 0}K\left(A\right)=\frac{N}{1+N\;\left(N-1\right)\;\left(N^{2}-2\right)}<{\text{lim}}_{A\to 0}\text{MCC}\left(A\right)=\frac{1}{\left(N-1\right)\;\left(N^{2}-2\right)}<1\;$,
5. MCC(*A*) *is monotonically decreasing, while* K(A) *is not. Indeed*, K(A) *is a convex function of A, achieving the global minimum, which is a negative value, when* $A=1+N\sqrt{N(N-1)}$.
6. *The divergence between* MCC(*A*) *and* K(A) *increases monotonically as A* → ∞.

**Fig 5:**
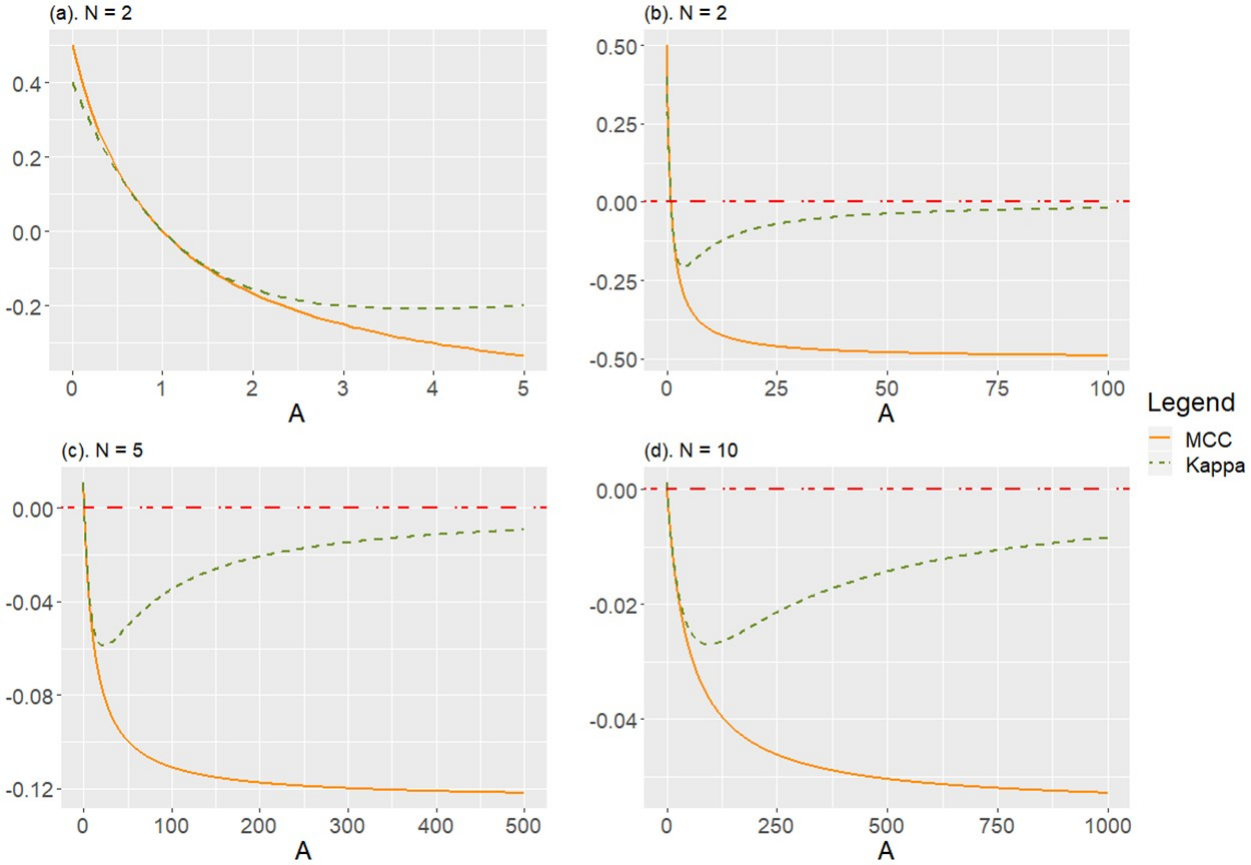
Disagreement between MCC and *Kappa* for *Z*_*A*_, for different values of *N*. (a) *N* = 2, a zoom of the detail for *A* ≤ 5. (b) *N* = 2, *A* ≤ 100. (c) *N* = 5, *A* ≤ 500. (d) *N* = 10, *A* ≤ 1000.

Fig 5 shows the behaviour of MCC and *Kappa* as functions of *A*, in cases *N* = 2 (both for *A* ≤ 5 and for *A* ≤ 100), and for *N* = 5 and *N* = 10. A desirable property of any measure of performance is its internal coherence, which implies that if the classifier moves gradually towards a worsening of the classification process, as is the case when *A* increases for the family *Z*_*A*_, the measure must reflect this fact with the consequent monotonous decrease (or increase, depending on the interpretation of the measure). Fig 5 highlights the incoherent behaviour of *Kappa*, since as we monotonically increase *A*, it does not exhibits a monotonic decreasing (as MCC does), and this anomaly not only happens in the binary case (*N* = 2), but continues to occur when we increase *N* above 2, although at a different scale. Therefore, we have seen that MCC shows internal coherence, unlike *Kappa*, which after decreasing in accordance with the worsening of the classification by increasing A, shows a monotonic growth that goes just in the opposite direction by continuing to increase *A*, which is clearly inconsistent.

## Experimental results

If we recapitulate, we have seen that both in the binary case with *c* = 1, and with the multidimensional *Z*_*A*_ family, as the asymmetry of the confusion matrix increased (*b* → +∞ and *A* → +∞, respectively), while its diagonal stays constant, the behaviour of *Kappa* and MCC differed more and more. This would be in line with the proven fact that if there is perfect symmetry, therefore these measures match (Proposition 1). It seems natural to ask if it is only the asymmetry that plays a determining role in the discrepancy observed in their linked behaviour (it seems that it should not be like that, since asymmetry of matrix *C*_0_ also increases as *b* → +∞, and yet the behaviour of *Kappa* and MCC agree). Or, on the contrary, there is any other characteristic of the matrix that drives in this circumstance. To try to shed some light on this issue, we have carried out some empirical experimentation in dimension *N* = 3.

We start by introducing a measure of the asymmetry of a matrix $M={\left(M_{ij}\right)}_{i,j=1}^{N}$, say *Asy*(*M*), by means of the Frobenius norm of the difference between the matrix and its transpose. That is to say, we define

$$\begin{array}{c} Asy\left(M\right)=||M-M^{T}\left|\right|=\sqrt{\sum_{i,j=1}^{N}{\left(M_{ij}-M_{ji}\right)}^{2}}\;. \end{array}$$

**Fig 6:**
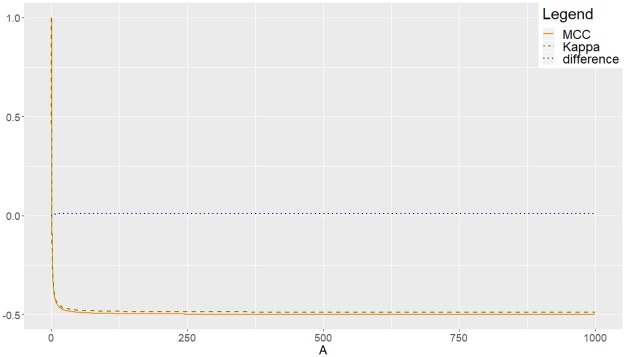
Experimental agreement between MCC and *Kappa* for *M*_1_(*A*). Increasing asymmetry but constant entropy.

**Example (a)** Let us consider matrix $M_{1}\left(A\right)=(\begin{array}{ccc} 1 & 2\;A & A\\ A & 1 & 2\;A\\ A & A & 1 \end{array})$, with *A* ≥ 1. Obviously, *M*_1_(*A*) is not symmetric, with *Asy*(*M*_1_(*A*)) = 2*A*, which increases with *A*, achieving the minimum = 2 when *A* = 1. We can make a graph showing the evolution of *Kappa* and MCC when increasing *A*, as shows Fig 6, where it can be observed that the behaviour of *Kappa* is very similar to that of MCC. Then, asymmetry has not been enough to generate a different behaviour of them. What, then?

Think about the entropy generated by the values of the matrix that are outside the main diagonal. In general, given a set of non-negative numbers, say {*n*_1_, …, *n*_*r*_}, the Shannon’s entropy generated by the set can be defined by $Ent=\sum_{i=1}^{r}-p_{i}\;\text{log}\left(p_{i}\right)$, with $p_{i}=\frac{n_{i}}{n}$ if $n=\sum_{i=1}^{r}n_{i}$, where log usually denotes logarithm in base 2. With this definition, *Ent*(*M*_1_(*A*)) = *Ent*({2*A*, *A*, *A*, 2*A*, *A*, *A*}) = 2.5, which is independent of *A*, so for the family of matrices *M*_1_(*A*), entropy can not play any role since it remains constant when *A* varies. The same happens with matrix *C*_0_, for which asymmetry increases as *b* → +∞ but entropy remains constant. In other words: increasing asymmetry but constant entropy does not produce the phenomenon of inappropriate behaviour of *Kappa* in which we are interested.

**Fig 7:**
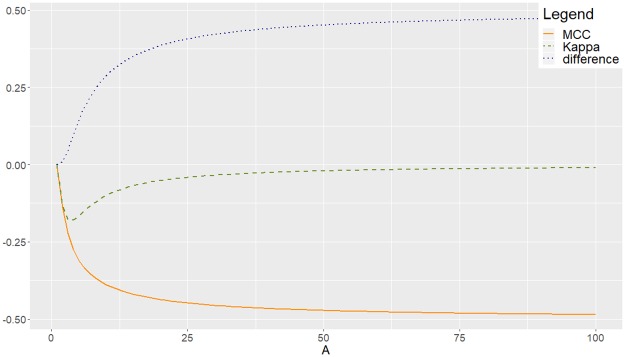
Experimental disagreement between MCC and *Kappa* for *M*_2_(*A*). Decreasing to zero entropy, which implies increasing asymmetry.

**Example (b)** Consider now matrix $M_{2}\left(A\right)=(\begin{array}{ccc} 1 & A & 1\\ 1 & 1 & A^{2}\\ 1 & 1 & 1 \end{array})$ with *A* > 1. Then $Asy\left(M_{2}\left(A\right)\right)=\sqrt{2}\;\left(A-1\right)\sqrt{1+{\left(A+1\right)}^{2}}\;$, which increases with *A*, and $\begin{array}{c} Ent\left(M_{2}\left(A\right)\right)=Ent\left(\left\{A,\;1,\;1,\;A^{2},\;1,\;1\right\}\right)=\text{log}\left(A\;\left(A+1\right)+4\right)-\frac{A\;(2\;A+1)}{A\;(A+1)+4}\;\text{log}\left(A\right) \end{array}$ decreases, converging to 0 as *A* → +∞. The corresponding plots of *Kappa*, MCC and the difference, with respect to *A* are shown in Fig 7.

MCC(*M*_2_(*A*)) is a decreasing function of *A* but $K(M_{2}\left(A\right))$ is increasing for *A* ≥ 4. Then, we can observe a contradictory behaviour of the two measures. Let us see this with numerical examples in Table 1: as *A* increases (and then, asymmetry increases while entropy decreases to zero), MCC decreases but *Kappa* increases.

**Table 1:** Comparing MCC, *Kappa*, *Asy* and *Ent* for *M*_2_(*A*). *A* = 10, 25, 50, 75, 100.

*M*_2_(*A*)	*A* = 10	*A* = 25	*A* = 50	*A* = 75	*A* = 100
MCC	-0.3879	-0.4478	-0.4722	-0.4810	-0.4856
*Kappa*	-0.1002	-0.0410	-0.0203	-0.0135	-0.0101
*Asy*	140.5845	883.1217	3534.7990	7954.2260	14141.4100
*Ent*	0.7135	0.2998	0.1590	0.1108	0.0859

**Remark 4** *Note that for matrix M*_2_(*A*), MCC *and Kappa diverge as A increases, as it happens with the family of matrices Z*_*A*_ *and with the confusion matrix* $C_{1}=(\begin{array}{cc} a & b\\ 1 & d \end{array})$ *considered in Proposition 3 (binary case with c* = 1 *in which the behaviour of Kappa appears as contrary to common sense when b increases). In the three scenarios, entropy decreases to zero and the asymmetry of the confusion matrix grows to* +∞. *Indeed, for matrices Z*_*A*_ (*as A* → +∞) *and C*_1_ (*as b* → +∞) *we have that*

$$\begin{array}{cc} Asy(Z_{A}) & =\sqrt{2}\;\left(A-1\right)↗+\infty \;,\\ Ent(Z_{A}) & =\text{log}\left(N^{2}-1+A\right)-\frac{A}{N^{2}-1+A}\;\text{log}\left(A\right)↘0\;,\\ Asy(C_{1}) & =\sqrt{2}\;\left|b-1\right|↗+\infty \;,\\ Ent(C_{1}) & =Ent\left(\left\{1,b\right\}\right)=\frac{-b}{b+1}\;\text{log}\left(b\right)+\text{log}\left(b+1\right)↘0\;. \end{array}$$

In general, entropy of the elements outside the main diagonal and asymmetry are related in the sense given by the following lemma.

**Lemma 9** *Let C*(*A*) = (*C*_*ij*_(*A*))_*i*,*j*=1,…,*N*_ *be a matrix of non-negative integers depending on a parameter A* ∈ ℕ, *and such that Ent*(*C*(*A*)) > 0 *for any A. Therefore, if the entropy of C*(*A*) *decreases to zero, asymmetry must grow to infinity, that is*,

$$\begin{array}{c} \underset{A\to +\infty }{\text{lim}}Ent\left(C\left(A\right)\right)=0\;\Rightarrow \;\underset{A\to +\infty }{\text{lim}}Asy\left(C\left(A\right)\right)=+\infty \;. \end{array}$$

*Proof*: By definition of Shannon’s entropy, if *Ent*(*C*(*A*)) converges to zero, then in the limit there is no uncertainty outside the main diagonal, that is, there must exist a pair (*i*, *j*), with *i* ≠ *j*, such that

$$\begin{array}{c} \underset{A\to +\infty }{\text{lim}}C_{ij}\left(A\right)=+\infty \;\text{and}\;\forall \left(r,s\right)\neq \left(i,j\right),\;\;\underset{A\to +\infty }{\text{lim}}\frac{C_{rs}\left(A\right)}{C_{ij}\left(A\right)}=0\;. \end{array}$$

Then, with (*r*, *s*) = (*j*, *i*), we can write

$$\begin{array}{c} \underset{A\to +\infty }{\text{lim}}(C_{ij}\left(A\right)-C_{ji}\left(A\right){)}^{2}=\underset{A\to +\infty }{\text{lim}}(1-\frac{C_{ji}\left(A\right)}{C_{ij}\left(A\right)}{)}^{2}\;C_{ij}^{2}\left(A\right)=+\infty \end{array}$$

since ${\text{lim}}_{A\to +\infty }(1-\frac{C_{ji}\left(A\right)}{C_{ij}\left(A\right)}{)}^{2}={\left(1-0\right)}^{2}=1$ and ${\text{lim}}_{A\to +\infty }C_{ij}^{2}\left(A\right)=+\infty$.

Finally, from the fact that *Asy*(*C*(*A*)) ≥ |*C*_*ij*_(*A*) − *C*_*ji*_(*A*)| → +∞ we finish the proof.

Lemma 9 confirms that what we have observed in different examples (confusion matrices *C*_1_ as function of *b*, *Z*_*A*_ and *M*_2_(*A*)), in which entropy tended to zero and asymmetry grew towards infinity, is not a coincidence but the rule.

It is still necessary to ask whether the role of asymmetry in observing the phenomenon of the discrepancy between the behaviours of *Kappa* and MCC is canceled out by entropy. That is, if the phenomenon still can be observed if the asymmetry remains constant while the entropy does not decrease to zero. The negative answer is given by the following example, in which asymmetry is constant and entropy decreases to a positive limit but the phenomenon of discrepancy between MCC and *Kappa* is no longer observed.

**Fig 8:**
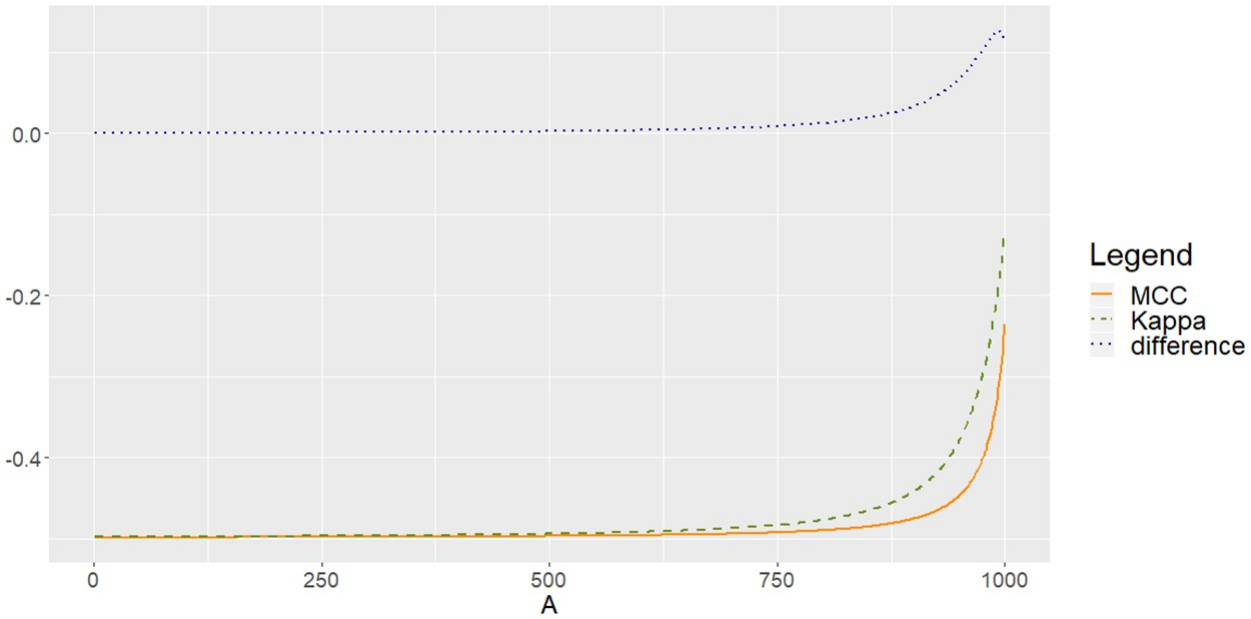
Experimental agreement between MCC and *Kappa* for *M*_3_(*A*). Decreasing entropy to a positive limit and constant asymmetry.

**Example (c)** Be matrix $M_{3}\left(A\right)=(\begin{array}{ccc} 1 & B & B\\ B+100 & 1 & B\\ B+100 & B+100 & 1 \end{array})$ with *B* = 1000 − *A*, *A* = 0,…, 999. The corresponding plot of MCC, *Kappa* and the difference in absolute value is shown in Fig 8. In this setting, as with Example (a), there is an agreement in the behaviour of MCC and *Kappa*. However, in this case there is no decrease of entropy to zero as in Example (b). Indeed, $Ent\left(M_{3}\left(A\right)\right)=\text{log}\left(6\;B+300\right)-\frac{1}{6\;B+300}\;(3\;B\;\text{log}(B)+3\;(B+100)\;\text{log}(B+100))$ with *B* = 1000 − *A*, is a monotonically decreasing function of *A* that converges to log(300) − log(100) > 0 as *A* → 1000, while $Asy\left(M_{3}\left(A\right)\right)=100\;\sqrt{6}$ remains constant.

Previous examples, in which the diagonal stays constant, show that it is not enough that the asymmetry grows to infinity, or that the entropy is constant or simply decreasing, for the phenomenon of discrepancy between *Kappa* and MCC to occur, but heuristically it seems that entropy must decrease to zero, which implies that at the same time asymmetry grows to infinity by Lemma 9. At least it is what experimentation has shown in the cases already commented. To finish, two more examples in the same vein, the first corresponding to the situation of discrepancy, and the latter to the similarity, in the behaviours of MCC and Kappa.

**Fig 9:**
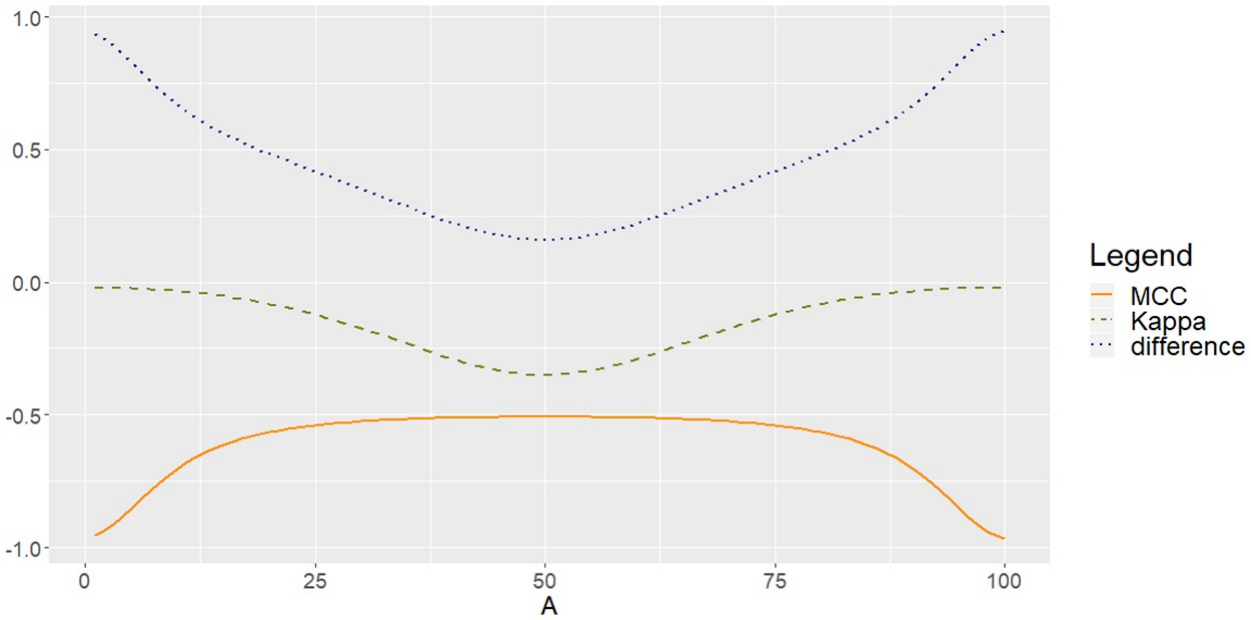
Experimental disagreement between MCC and *Kappa* for *M*_4_(*A*). Entropy decreases to zero, which implies that asymmetry increases, for *A* increasing from 50 to 100, and from *A* decreasing from 50 to 0, by symmetry.

**Example (d)** Let be the confusion matrix $M_{4}\left(A\right)=(\begin{array}{ccc} 1 & A & 1\\ A^{2} & 1 & B\\ 1 & B^{2} & 1 \end{array})$, with *B* = 100 − *A* and *A* = 50,…, 100. In this case, as function of *A* ∈ [50, 100],

$$\begin{array}{c} Asy\left(M_{4}\left(A\right)\right)=\sqrt{2}\;\sqrt{A^{2}\;{\left(A-1\right)}^{2}+{\left(100-A\right)}^{2}\;{\left(99-A\right)}^{2}} \end{array}$$

monotonically increases with *A*, and

$$\begin{array}{c} Ent\left(M_{4}\left(A\right)\right)=\text{log}\;\left(g\left(A\right)\right)-\frac{A(2A+1)\;\text{log}\;(A)+(100-A)(201-2\;A)\;\text{log}\;(100-A)}{g(A)}\;, \end{array}$$

with *g*(*A*) = *A*(*A* + 1) + (100 − *A*)(101 − *A*) + 2, monotonically decreases (to zero if we increase the parameter 100). We can observe in Fig 9 that in this case the appearance of the described phenomenon of behaviour against the common sense of *Kappa* is confirmed: for *A* > 50, MCC decreases and *Kappa* increases as *A* increases. By symmetry, for *A* < 50 we observe just the same when *A* decreases.

Table 2 illustrates this example numerically through a particular case in which we compare different values of *A*. We observe that when entropy decreases and asymmetry increases (*A* > 50) MCC decreases and *Kappa* increases, while a completely symmetrical behaviour is observed for *A* < 50, according to Fig 9.

**Table 2:** Comparing MCC, *Kappa*, *Asy* and *Ent* for *M*_4_(*A*). *A* = 50, 60, 70, 80, 90, 100.

*M*_4_(*A*)	*A* = 50	*A* = 60	*A* = 70	*A* = 80	*A* = 90	*A* = 100
MCC	-0.5081	-0.5114	-0.5249	-0.5653	-0.7032	-0.9659
*Kappa*	-0.3500	-0.2900	-0.1735	-0.0817	-0.0341	-0.0200
*Asy*	4900.0000	5470.868	6940.576	8953.971	11328.5700	14000.7100
*Ent*	1.1442	1.0319	0.7554	0.4418	0.1970	0.0830

**Example (e)** Let be the confusion matrix $M_{5}\left(A\right)=(\begin{array}{ccc} 1 & 2\;A & A\\ A & 1 & A+100\\ A & A & 1 \end{array})$. As function of *A* ≥ 1, $Asy\left(M_{5}\left(A\right)\right)=\sqrt{2}\;\sqrt{A^{2}+100^{2}}↗+\infty$ and is increasing, while

$$\begin{array}{c} Ent\left(M_{5}\left(A\right)\right)=\text{log}\left(7\;A+100\right)-\frac{8\;A\;\text{log}(A)+(A+100)\;\text{log}(A+100)}{7\;A+100} \end{array}$$

decreases to log(7) − 2/7 > 0 when *A* → +∞. In this case, MCC and *Kappa* agree in behaviour as *A* increases.

## Conclusion

Accuracy is one of the most intuitive and widely used performance metrics for classification although it is not appropriate when considering unbalanced cases. MCC and *Kappa* seem to correct this bias: the former was initially designed to deal with very unbalanced data, while the latter, which was not created to be a classification performance metric but that, however, is widely used for this, takes into account the probability of getting the classification by pure chance. These two measures have a similar behaviour in some situations. In fact, we show that they coincide precisely when the confusion matrix is perfectly symmetric. In other situations, however, their behaviour can diverge to the point that *Kappa* should be avoided as a measure of behaviour to compare classifiers in favor of more robust measures as MCC.

In the present work, similarities and differences among MCC and *Kappa* have been discussed and illustrated with synthetic confusion matrices, both in the binary and in the multi-class setting. Our mathematical analysis and heuristic study show that in situations in which the diagonal of the confusion matrix stays constant and at the same time there is a decrease to zero of the entropy of the elements outside the diagonal, which implies an increase in the asymmetry of the confusion matrix, the phenomenon of qualitative differentiation in the behaviour of *Kappa* and MCC appears clearly. Notwithstanding, neither increasing nor constant asymmetry when entropy is not decreasing to zero, does not seem to be enough to produce this phenomenon. As far as we know, this kind of conclusions have not been reached before, so they represent a novelty in the study of *Kappa*.

From a clinical perspective, the fact that *Kappa* is a relative measure of agreement is problematic since it is hard to set a threshold for a good agreement. This does not seem to be a problem when it is used as a performance metric, because *Kappa* values are compared for each classifier given a unique ground-truth, being the relative difference and not the value itself, which determines the best classifier. Notwithstanding, we have shown that if marginal probabilities are really small, the distribution of the misclassification also affects the value of *Kappa*, to the extent that worse classification results can obtain, however, higher values of the statistic. This is especially dramatic when the entropy of the elements outside the main diagonal of the confusion matrix decreases to zero.

A summary of the examples that have been considered in this work according to the agreement/disagreement between the behaviour of MCC and *Kappa*, can be found in the Table 3.

**Table 3:** Summary of the obtained results: Examples and agreement/disagreement between the behaviour of MCC and *Kappa* in terms of the asymmetry of the confusion matrix and of the entropy associated to the elements outside the main diagonal. Disagreement scenario corresponds to entropy decreasing to zero, which implies by Lemma 9 that asymmetry must grow to infinity.

	Asymmetry ↗ +∞	Asymmetry = constant
Entropy = constant	Agreement *C*_0_, *M*_1_(*A*)	
Entropy ↘ 0	Disagreement *C*_1_, *Z*_*A*_, *M*_2_(*A*), *M*_4_(*A*)	
Entropy ↘ > 0	Agreement *M*_5_(*A*)	Agreement *M*_3_(*A*)

The standard problems associated with *Kappa* are mainly related to unbalanced datasets (see for instance [36] and [17]). We show that an unbalanced situation can make *Kappa* not comparable between different situations, but to achieve counter-intuitive results, it is also necessary that the entropy of the elements outside the main diagonal to decrease to zero.

Nowadays, in the field of machine learning such situations, in which the number of observations of one of the classes far exceed the quantity of the others, or when the marginal distributions are small, are very common. Machine learning algorithms automatically scrutinize huge amount of data, classifying it into hundreds of categories or look for an unlikely relevant event. In that framework, the finding of a dependable performance measure to be robust and reliable becomes of the utmost importance. Hence, we believe that it has been sufficiently justified that, unfortunately, Cohen’s *Kappa* can no longer play this role, especially considering the existence of solid alternatives.
